# Altered sleep behavior in a genetic mouse model of impaired fear extinction

**DOI:** 10.1038/s41598-021-88475-2

**Published:** 2021-04-26

**Authors:** Eva Maria Fritz, Matthias Kreuzer, Alp Altunkaya, Nicolas Singewald, Thomas Fenzl

**Affiliations:** 1 Department of Pharmacology and Toxicology, Institute of Pharmacy and CMBI, University of Innsbruck, Innsbruck, Austria; 2 Department of Anesthesiology and Intensive Care, School of Medicine, Klinikum Rechts Der Isar, Technical University of Munich, Ismaninger Straße 22, 81675 Munich, Germany

**Keywords:** Anxiety, Post-traumatic stress disorder, Psychiatric disorders, Diseases, Neuroscience, Circadian rhythms and sleep, Learning and memory

## Abstract

Sleep disturbances are a common complaint of anxiety patients and constitute a hallmark feature of post-traumatic stress disorder (PTSD). Emerging evidence suggests that poor sleep is not only a secondary symptom of anxiety- and trauma-related disorders but represents a risk factor in their development, for example by interfering with emotional memory processing. Fear extinction is a critical mechanism for the attenuation of fearful and traumatic memories and multiple studies suggest that healthy sleep is crucial for the formation of extinction memories. However, fear extinction is often impaired in anxiety- and trauma-related disorders—an endophenotype that is perfectly modelled in the 129S1/SvImJ inbred mouse strain. To investigate whether these mice exhibit altered sleep at baseline that could predispose them towards maladaptive fear processing, we compared their circadian sleep/wake patterns to those of typically extinction-competent C57BL/6 mice. We found significant differences regarding diurnal distribution of sleep and wakefulness, but also sleep architecture, spectral features and sleep spindle events. With regard to sleep disturbances reported by anxiety- and PTSD patients, our findings strengthen the 129S1/SvImJ mouse models’ face validity and highlight it as a platform to investigate novel, sleep-focused diagnostic and therapeutic strategies. Whether the identified alterations causally contribute to its pathological anxiety/PTSD-like phenotype will, however, have to be addressed in future studies.

## Introduction

‘Sleeping your worries away’ is not just a hollow phrase: an abundance of evidence approves that healthy sleep is crucial for emotional coping^1,2^. One central mechanism in coping with and overcoming fearful or even trauma-eliciting situations is fear extinction, during which conditioned fear responses are attenuated by the formation of new, fear-inhibitory memories^3,4^. This process is also the cornerstone of exposure-based cognitive behavioral therapy, which is commonly employed in anxiety- and trauma-related disorders^5^. However, not all patients respond well to the treatment and in many, fear returns after an initially successful intervention^6,7^. A possible reason may be individual deficits in the ability to acquire, consolidate or retrieve extinction memories, which influence treatment outcomes^8,9^. Indeed, impaired fear extinction has been reported in anxiety-related psychiatric conditions and particularly in post-traumatic stress disorder (PTSD)^10–12^. Results from multiple studies in humans suggest that healthy sleep plays an important role in the formation of extinction memories, which led to the hypothesis that disturbed sleep could underlie the inability to extinguish fear^13–15^. Complaints about disrupted sleep are very common among patients suffering from anxiety-related disorders (for review see Ref.^16^). In PTSD, sleep disturbances even have the status of a hallmark feature, with up to 90% of patients reporting poor subjective sleep quality, nightmares and insomnia^17,18^. Subjective sleep complaints are partly corroborated by polysomnographic studies, which have delivered evidence of disturbed sleep in PTSD and other anxiety disorders, but were unable to define single sleep parameters that were exclusively altered in these disorders (for meta-analyses see Refs.^19,20^. In the case of PTSD, several prospective studies convincingly demonstrated that sleep disturbances occurring prior to or shortly after trauma exposure can be a predictor of poor psychiatric outcomes (e.g. Refs.^21–23^, for meta-analysis see Ref.^24^). Also, individuals reporting sleep disturbances at baseline were more likely to develop a panic disorder or generalized anxiety disorder later in life^25^. Interestingly, interventions specifically targeting sleep disruptions in anxiety and PTSD patients were able to improve daytime symptoms^26,27^. Persisting sleep problems, however, were reported to interfere with remission from PTSD^28^. Therefore, it has been hypothesized that impaired sleep may represent more than just a secondary symptom of anxiety- and trauma-related disorders, but rather a predisposing, precipitating and perpetuating factor in disease development^29–31^.

Rodent models are an indispensable tool in studying the biological underpinnings of human psychiatric disease and investigating novel biomarkers and treatments. Modeling specific endophenotypes that represent the core features of the disease has become increasingly relevant since the rise of the research domain criteria (RDoC) initiative^32^. Impaired fear extinction is considered a robust clinical endophenotype of anxiety- and trauma-related disorders, and the 129S1/SvImJ (S1) inbred mouse strain, which exhibits a profound inability to extinguish fear and consolidate extinction memories when compared to normally extinguishing mouse strains [e.g. C57BL/6 (BL6)]^33,34^, represents a prototype of this clinical feature^35^. The maladaptive fear phenotype of the S1 mouse strain is furthermore characterized by deficits in safety learning and high susceptibility to fear generalization to ambiguous contexts or cues^36,37^, which are also characteristics of anxiety-related disorders^38,39^. Moreover, compared to BL6, S1 mice show an anxiety-like phenotype as well as reduced novelty- and reward seeking and an altered neuroendocrine stress response^40^. Aberrant neuronal activation patterns of fear- and extinction-relevant brain regions in S1 mice reported in immediate early gene mappings^33,41^ as well as ex-vivo and in-vivo electrophysiological recordings^42,43^ suggest a failure to properly engage the corticolimbic extinction circuitry: while activity in pro-extinction areas like infralimbic cortex and basolateral amygdala is typically reduced, increased activity is detectable in pro-fear regions like prelimbic cortex and the central medial nucleus of the amygdala. This corresponds very well to imaging data from PTSD patients which typically show a hypoactivation of the ventromedial prefrontal cortex, the human equivalent to the infralimbic cortex, and an exaggerated amygdala reactivity during extinction retrieval^44,45^.

In the present study, we report a profoundly altered sleep/wake behavior in the S1 mouse strain as compared to sleep/wake profiles of typically extinction-competent BL6 mice for the first time. Our findings strengthen the face validity of the S1 mouse model and substantiate its relevance as a platform for future research on prognostic and diagnostic sleep biomarkers as well as sleep-targeted interventions in anxiety- and trauma-related disorders.

## Methods

### Animals and housing conditions

Animal care and experiments were carried out in compliance with national and international guidelines for animal welfare and as approved by the Austrian Bundesministerium für Wissenschaft und Forschung (BMWF-66.008/0011-WF/V/3b/2014). The study was approved by the ethics committee of the Austrian Bundesministerium für Wissenschaft und Forschung and carried out in compliance with the ARRIVE guidelines. Male C57BL/6N mice (BL6; n = 8) and 129S1/SvlmJ mice (S1; n = 8) were bred in-house and transferred from the animal facility to the laboratory a few days before surgery, at an age of 11–18 weeks. All animals included in the study were assessed for regular home cage behavior and EEG parameters. We excluded one BL6 animal from all analyses due to abnormal behavior after surgery (uni-directional right-handed hyper-locomotion). In the laboratory, all mice were housed in individual home cages (side-by-side) in sound-attenuated chambers (custom-made, M. Streicher, Innsbruck, Austria) under a constant light/dark cycle (12 h/12 h; lights ON at 10 a.m.) and at a constant temperature (24 °C ± 1 °C). All home cages (26 × 26 × 35 cm) had clear Lucite walls, an open top side (custom-made, M. Streicher, Innsbruck, Austria) and contained wooden chips and torn tissue paper as bedding and nesting material. Water and food were supplied ad libitum.

### Surgical procedure and electrode manufacturing

We carried out surgery and electrode manufacturing as described previously^46,47^. All surgical procedures were performed under isoflurane anesthesia (1.9–2.2% isoflurane, air flow rate 190–200 ml/min; Univentor 410 Anesthesia Unit, AgnTho's, Lidingö, Sweden) provided through a custom-made breathing mask while the animal was head-fixed in a stereotaxic frame (Leica Angle Two, Leica Biosystems, Wetzlar, Germany). Meloxicam was administered subcutaneously as peri-operative analgesic (0.5 mg/kg; Metacam, B. Braun Melsungen, Melsungen, Germany). At a sufficient level of anesthesia, the scalp was opened by a rostrocaudal incision and, using a dental precision driller (Typ 4811, KaVo, Biberach, Germany), six craniotomies (ø 200 μm) were made bilaterally along the sagittal suture. Two jeweler's screws (ø 150 μm) were mounted into the central holes to build a fundament using dental cement (Paladur, Heraeus-Kulzer, Hanau, Germany) and ensure fixation of the socket board to the skull. We manufactured the socket boards, to which the headstage recording cable was attached later, customly by soldering six gold-wire electrodes (ø 150 µm; Häfner, Leopoldshöhe, Germany) with ball-shaped ends onto an 8-pin PCB connector (Type 861-87-008-10-001101, preci-dip, Delémont, Switzerland). Four epidural electrodes were then inserted via the anterior and posterior craniotomies, i.e. two electroencephalography (EEG) electrodes under the frontal bone (from Bregma: AP + 1.0 mm, ML ± 1.0 mm) and ground and reference electrodes under the parietal bone (from Bregma: AP − 1.8 mm, ML ± 1.0 mm). Two electromyography (EMG) electrodes were inserted bilaterally into the neck muscle caudal to the occipital bone. For proper isolation of the electrodes and better durability of the implant, the socket board was thoroughly encased in dental cement. Finally, the wound was sutured (PERMA-HAND Silk Suture C-1/5-0, Ethicon, Bridgewater, NJ, USA) around the implant. To reduce post-operative pain, Meloxicam was provided via drinking water (5 µg/ml; Metacam, B. Braun Melsungen, Melsungen, Germany) for the following 7 days and we allowed all animals to recover for a total of 14 days before starting recording sessions.

### Recording setup and experiment protocol

We performed all electrophysiological recordings as described previously^47^. The implanted socket boards served as connection to the recording system via a headstage recording cable containing a pre-amplifier (amplification factor 1×; custom-made, npi electronics, Tamm, Germany) which in turn was attached to a commutator (SL-10 slip-ring commutator, Dragonfly Research & Development, Ridgeley, WV, USA). To minimize restraint of the animals by the recording cable, the commutator was balanced on a 3-dimensionally moving, weight-balanced swivel (custom-made, M. Streicher, Innsbruck, Austria), allowing them to move freely within their home cages. All mice were habituated to the recording chamber for 7 days and to the recording cable for 3 days before start of the recording sessions. All recordings were performed in an electrically shielded (bench-top Faraday cage, TMC, Peabody, MA, USA) sound-attenuated chamber (custom-made, M. Streicher, Innsbruck, Austria), in which the individual home cages were placed in pairs of two. The signals of all electrodes were fed into individual amplifier units (amplification factor 1000×; Type DPA-2FL, npi electronics, Tamm, Germany) and hardware band pass filtered (EEG: 0.1–100 Hz, EMG: 1–100 Hz). Each signal was digitized at a sampling rate of 250 Hz (Power 1401-3 AD-board, Cambridge Electronic Design Limited, Cambridge, UK), recorded and stored for offline analysis using Spike2 Software (Cambridge Electronic Design, Cambridge, UK). We performed at least three recording sessions per animal on consecutive days, each starting at the beginning of the light period and lasting for 23 h (10 a.m. to 9 a.m.). The 1 h gap (9 a.m. to 10 a.m.) served for animal care and setup maintenance. To allow maximum habituation of the animals to the recording setup we used the last recording session for data analysis, while the other sessions served as backups in case of technical issues.

### Data processing and sleep scoring

We performed data processing routines as described previously^47,48^ using custom-written scripts for MATLAB (Mathworks, Natick, MA, USA). Raw recording files were exported from Spike2 as spreadsheets from which EEG and EMG data were extracted separately and down sampled to 125 Hz after appropriate filtering. The resulting vectors were then imported into a LABVIEW-based (National Instruments, Austin, TX, USA) semi-automated sleep scoring software developed by the authors^48^, which uses an algorithm developed for sleep analysis in rats^49^ adapted for mice^50^. Accordingly, the vigilance states wakefulness (WAKE), non-rapid eye movement sleep (NREMS) and rapid-eye movement sleep (REMS) were assigned to non-overlapping 4 s EEG episodes (1 epoch = 4 s). An experienced scorer blinded to the strain reviewed the randomized, semi-automated scoring vectors manually at epoch-level to ensure accuracy of vigilance state assignment at transitions. Vigilance state changes were re-scored in a way that resulting bouts lasted longer than 3 epochs (12 s). The derived rescored vectors were then exported in spreadsheet format and used for all further quantitative and qualitative analyses.

### Quantitative EEG analysis

We analyzed EEG/EMG data of all single animals for several different parameters. For quantitative sleep/wake analyses, we calculated the mean percentages of the vigilance states WAKE, NREMS and REMS as 2 h means across the whole recording session (23 h) and also bins for light (inactive; 12 h) and dark (active; 11 h) period. To evaluate sleep architecture and fragmentation, we used custom-written MATLAB scripts to graph hypnograms and determine transitions between all vigilance states as well as bout numbers and bout lengths for light and dark period separately. In our opinion, the simple occurrence of the very first NREMS or REMS bout in the inactive period does not properly reflect NREMS or REMS latency in fragmented rodent sleep patterns, so we calculated NREMS and REMS latency as mean time until the occurrence of the first 5 NREMS or REMS bouts (with one bout corresponding to at least 12 s of consecutive NREMS or REMS) in the light period.

### Spectral EEG analysis

Power spectral density (PSD) for WAKE, NREMS and REMS during light and dark period was calculated using the MATLAB *pwelch* function with a frequency resolution of 0.25 Hz for non-overlapping 4 s EEG epochs synchronized to the respective vigilance state scoring results. Before PSD calculation we applied a 1 Hz high-pass filter using the MATLAB *filtfilt* function to reduce movement-associated artifacts at low frequencies. For following analyses, we normalized PSD values by dividing the power for each 0.25 Hz frequency bin by the total power of the respective EEG epoch. Analogous to previous publications^46–48,50^, we considered the following frequency bands for analysis: delta (δ, 1.0–5.0 Hz), theta (θ, 6.0–9.0 Hz), alpha (α, 10.0–15.0 Hz) and eta (η, 16.0–22.75 Hz). We excluded two animals from the analysis of spectral features due to an unspecific attenuation of the PSD in high frequency ranges (η band). Nevertheless, these animals remained included in quantitative EEG and sleep spindle analyses as low frequency ranges were unaffected and visual rescoring on an epoch level did not reveal any abnormalities in EEG traces.

### Sleep spindle detection

We performed sleep spindle analysis using an automated MATLAB-based paradigm validated for the detection of spindles from mouse EEG recordings by Uygun et al.^51^. For this, raw EEG data and the corresponding rescored vectors for all animals were imported into the software and processed and automatically screened for sleep spindles as described^51^. Detection parameters were set to the following values as suggested by the authors: minimum spindle duration: 0.5 s, maximum spindle duration: 10 s, inter-spindle interval: 0.1 s. Also, all data processing steps were performed according to the recommendations by the authors^51^. In short, raw EEG traces were first bandpass filtered between 10 and 15 Hz to consider the peak spindle frequency of approximately 11 Hz in mice. For this, a Butterworth band-pass filter with the following characteristics was applied: first stopband frequency = 3 Hz, first passband frequency = 10 Hz, second passband frequency = 15 Hz, second stopband frequency = 22 Hz, stopband attenuation 24 dB/octave. The root mean square (RMS) of the filtered EEG traces was calculated using a 750 ms window to smoothen the trace and generate a signal envelope. RMS values were then cubed to enhance the signal-to-noise ratio on the y-axis and facilitate threshold definition. A two-threshold approach was used to establish inclusion criteria for spindle detection during NREMS (lower threshold: 1.0 mean cubed RMS; upper threshold: 2.5 mean cubed RMS; for details please refer to the original publication from Uygun and coworkers).

### Statistical analysis

All statistical tests were performed with GraphPad Prism 8 (GraphPad Software, San Diego, CA, USA) or MATLAB (Mathworks, Natick, MA, USA). For comparison of circadian sleep/wake behaviors between S1 and BL6 mice and sleep spindle numbers across 23 h, quantile–quantile (QQ) plots were used to assess normality (Supplementary Fig. S1). Then, a *two-way repeated measures (RM) ANOVA with one factor repetition* (factors: ‘strain’, ‘time’ = RM) was applied, followed by a *Sidak post-hoc test* for multiple comparisons (*p < 0.05, **p < 0.01, ***p < 0.001) in case significant main or interaction effects were detected. We used a *Mann–Whitney U test* (two-tailed; *p < 0.05, **p < 0.01, ***p < 0.001) for pairwise comparisons of 12 h vigilance state percentages, all parameters to characterize sleep architecture as well PSDs within defined frequency bands and 12 h sleep spindle measures. The 0.25 Hz-binned PSD values were not normally distributed, thus we refrained from using a parametric test and instead calculated the *area under the curve* (AUC) with 10 k-fold bootstrapped 95% confidence intervals (CI) [lower endpoint; upper endpoint] using the *MES toolbox* for MATLAB^52^. If the 95% CI of the AUC does not include 0.5, an observed effect is different from chance, i.e. significant. Furthermore, an AUC > 0.7 or < 0.3 (depending on the direction of the observation) can be considered as a relevant effect^53^. To strengthen the validity of our statistical analysis, we also supported the p-values of all *Mann–Whitney U tests* with AUC effect sizes.

## Results

### The diurnal distribution of sleep and wakefulness is fundamentally different in S1 and BL6 mice

**Figure 1:**
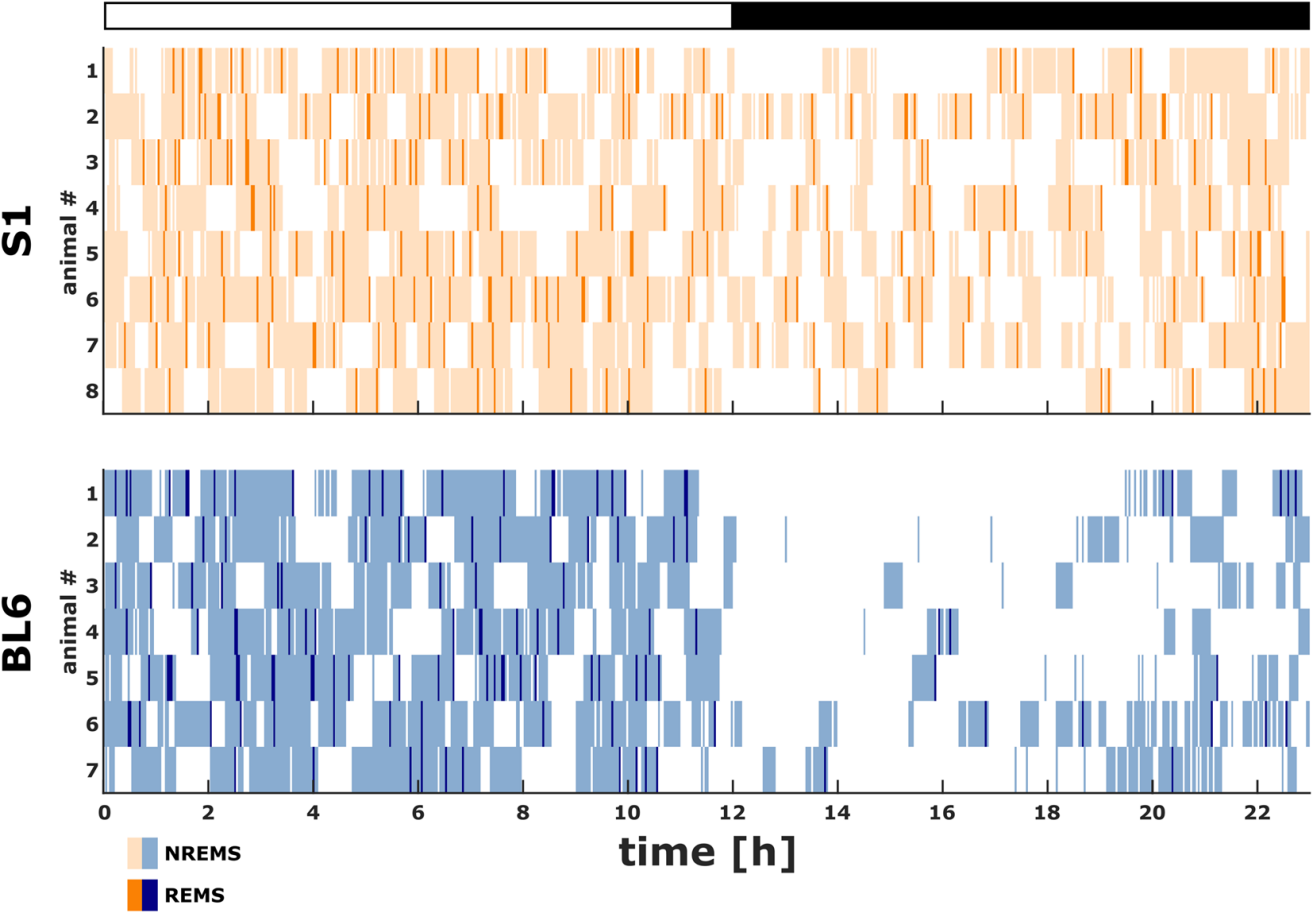
23 h hypnograms of S1 (n = 8) and BL6 (n = 7) mice. S1 mice displayed a less pronounced diurnal rhythmicity with more fragmented sleep/wake patterns and a higher amount of sleep intrusions in the dark period. White and black bars at the top represent light period (Zeitgeber time: h0–h12) and dark period (Zeitgeber time: h12–h23), respectively.

The hypnograms (Fig. 1) of all investigated animals provide an overview of the different circadian distribution of sleep and wakefulness as well as sleep fragmentation in S1 and BL6 mice. While BL6 animals demonstrated a clear diurnal rhythmicity that was coupled to the light–dark cycle, S1 mice exhibited a less prominent diurnal distribution of sleep and wakefulness with less consolidated periods of wakefulness during the dark period and more sleep intrusions.

### S1 mice spend significantly more time in NREMS and REMS during the dark (active) period than BL6 mice

**Figure 2:**
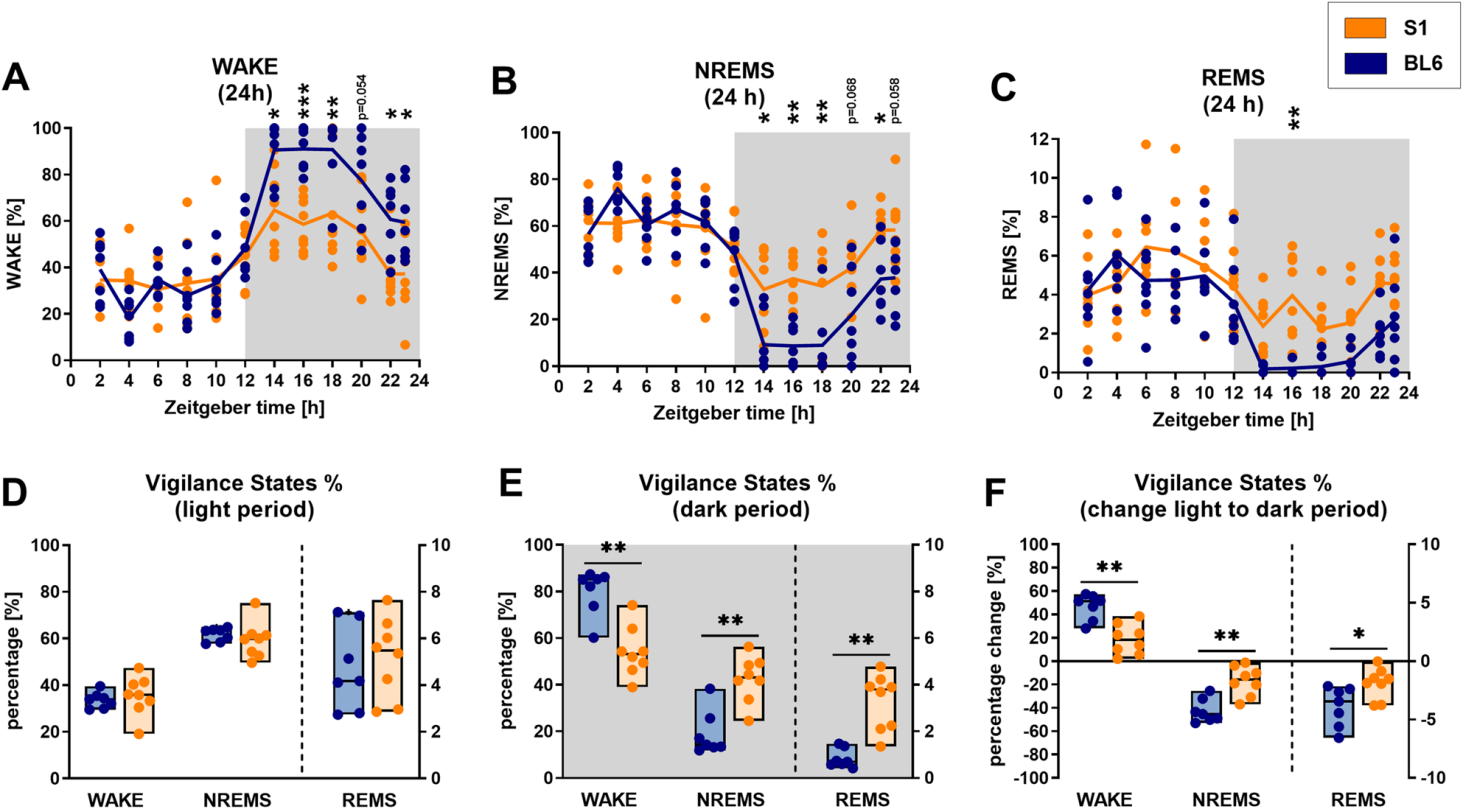
Circadian sleep/wake behavior of S1 (n = 8) compared to BL6 (n = 7) mice. The circadian time courses of all vigilance states differed between S1 and BL6 mice [(**A**) WAKE: ‘strain’: p = 0.002, ‘time’: p < 0.001, ‘time x strain interaction’: p < 0.001; (**B**) NREMS: ‘strain’: p = 0.003, ‘time’: p < 0.001, ‘time x strain interaction’: p < 0.001; (**C**) REMS: ‘strain’: p = 0.040, ‘time’: p < 0.001, time x strain interaction: p = 0.003]. In the light period (**D**), 12-h average percentages of all vigilance states were similar in S1 and BL6 mice (WAKE: p = 0.281; NREMS: p = 0.232; REMS: p = 0.463). Differences were mainly confined to the dark period (**E**), where S1 mice spent more time in NREMS (p = 0.002) and REMS (p = 0.001), while the percentage of WAKE (p = 0.002) was decreased. The diurnal change in the percentage of sleep and wakefulness from light to dark period (**F**) was less pronounced in S1 than BL6 animals (WAKE: p = 0.002; NREMS: p = 0.002; REMS: p = 0.021). (**A**–**C**) All graphs show 2 h means and individual data points, white and grey backgrounds indicate light and dark period. Statistical significance was determined by two-way RM ANOVA with one-factor repetition (Sidak post-hoc test). (**D**–**F**) All floating bars show min-to-max values with median and individual data points. Statistical significance was determined with a Mann–Whitney U test (*p < 0.05, **p < 0.01).

To quantitatively characterize the different sleep/wake phenotypes of S1 and BL6 mice, we calculated the amounts of sleep and wakefulness in both mouse lines across 23 h of recording (Fig. 2, for detailed statistics please see Tables 1 and 2 and Supplementary Fig. S1). S1 as well as BL6 mice showed circadian changes in sleep/wake activity that are typical for nocturnal animals, with higher amounts of sleep (NREMS, REMS) and less wakefulness (WAKE) during the light (inactive) period (Zeitgeber time: h0–h12) and vice versa during the dark (active) period (Zeitgeber time: h12–h23). However, the circadian distribution of all vigilance states (Fig. 2A–C) was significantly different in S1 compared to BL6 mice (‘strain’: WAKE: p = 0.002; NREMS: p = 0.003; REMS: p = 0.004 and ‘time x strain’: WAKE: p < 0.001; NREMS: p < 0.001; REMS: p = 0.003). While vigilance state percentages in the light period (Fig. 2D) were comparable in S1 and BL6 mice (WAKE: p = 0.281; NREMS: p = 0.232; REMS: p = 0.463), differences in sleep/wake behavior were mainly confined to the dark period (Fig. 2E). Here, S1 mice spent significantly more time sleeping, with increased amounts of NREMS (p = 0.002) and REMS (p = 0.001), while the percentage of WAKE (p = 0.002) was significantly reduced. Although both strains spent the majority of light and dark period in NREMS/REMS and WAKE, respectively, the diurnal changes between light and dark period (Fig. 2F) were less pronounced in S1 animals in all vigilance states (WAKE: p = 0.002; NREMS: p = 0.002; REMS: p = 0.021).

**Table 1:** Statistical results of Two-way RM ANOVAs with Sidak post-hoc test.

Figure		Two-way RM ANOVA				
		p	F	Sidak post-hoc (S1 vs. BL6)		
2A	Strain	0.002	F (1,13) = 15.06	Light period	Hours 2–12	p > 0.08
	Time	< 0.001	F (11,143) = 27.63	Dark period	Hour 14	p = 0.011
	Time × strain	< 0.001	F (11,143) = 4.53		Hour 16	p < 0.001
					Hour 18	p = 0.006
					Hour 20	p = 0.054
					Hour 22	p = 0.029
					Hour 23	p = 0.048
2B	Strain	0.003	F (1,13) = 15.06	Light period	Hours 2–12	p > 0.08
	Time	< 0.001	F (11,143) = 27.63	Dark period	Hour 14	p = 0.014
	Time × strain	< 0.001	F (11,143) = 4.53		Hour 16	p = 0.001
					Hour 18	p = 0.006
					Hour 20	p = 0.068
					Hour 22	p = 0.047
					Hour 23	p = 0.058
2C	Strain	0.004	F (1,13) = 5.23	Light period	Hours 2–12	p > 0.08
	Time	< 0.001	F (11,143) = 16.07	Dark period	Hour 14	p = 0.316
	Time × strain	0.003	F (11,143) = 2.74		Hour 16	p = 0.004
					Hour 18	p = 0.006
					Hour 20	p = 0.459
					Hour 22	p = 0.117
					Hour 23	p = 0.572
6B	Strain	0.0674	F (1,13) = 3.98	Light period	Hours 2–12	p > 0.08
	Time	< 0.001	F (11,143) = 26.12	Dark period	Hour 14	p = 0.033
	Time × strain	< 0.001	F (11,143) = 5.70		Hour 16	p = 0.003
					Hour 18	p = 0.006
					Hour 20	p = 0.018
					Hour 22	p = 0.101
					Hour 23	p = 0.999

**Table 2:** Statistical results of Mann–Whitney U tests and corresponding AUC with 95% CI.

Figure		Mann–Whitney U				AUC		
		Median (S1)	Median (BL6)	p	U	AUC	95% CI	
2D	WAKE	35.90, n = 8	33.92, n = 7	0.281	18.0	0.321	0.071	0.625
	NREMS	59.88, n = 8	63.28, n = 7	0.232	17.0	0.696	0.393	0.946
	REMS	5.49, n = 8	4.18, n = 7	0.463	21.0	0.375	0.089	0.696
2E	WAKE	53.11, n = 8	85.13, n = 7	0.002	3.0	0.946	0.795	1.000
	NREMS	43.08, n = 8	14.29, n = 7	0.002	3.0	0.054	0.000	0.196
	REMS	3.80, n = 8	0.70, n = 7	0.001	2.0	0.036	0.000	0.161
2G	WAKE	18.30, n = 8	51.37, n = 7	0.002	3.0	0.946	0.804	1,000
	NREMS	− 15.69, n = 8	− 45.17, n = 7	0.002	3.0	0.054	0.000	0.196
	REMS	− 1.69, n = 8	− 3.44, n = 7	0.021	8.0	0.143	0.000	0.375
3A		250.5, n = 8	240.0, n = 7	0.758	25.0	0.446	0.125	0.750
3B		176.0, n = 8	75.0, n = 7	0.009	6.0	0.107	0.000	0.304
3C	WAKE	59.5, n = 8	60.0, n = 7	0.979	27.5	0.491	0.179	0.813
	NREMS	120.0, n = 8	116.0, n = 7	0.676	24.0	0.429	0.152	0.741
	REMS	58.0, n = 8	66.0, n = 7	0.515	22.0	0.531	0.234	0.828
3D	WAKE	52.5, n = 8	34.0, n = 7	0.127	14.5	0.259	0.036	0.554
	NREMS	82.5, n = 8	35.0, n = 7	0.009	6.0	0.107	0.000	0.304
	REMS	43.0, n = 8	11.0, n = 7	0.001	1.0	0.018	0.000	0.107
3E	WAKE	3.464, n = 8	4.113, n = 7	0.613	23.0	0.589	0.268	0.893
	NREMS	4.050, n = 8	3.875, n = 7	0.955	27.0	0.482	0.125	0.839
	REMS	0.620, n = 8	0.493, n = 7	0.336	19.0	0.339	0.054	0.661
3F	WAKE	6.411, n = 8	16.89, n = 7	0.009	6.0	0.893	0.679	1.000
	NREMS	3.440, n = 8	2.695, n = 7	0.072	12.0	0.214	0.000	0.500
	REMS	0.597, n = 8	0.442, n = 7	0.121	14.0	0.250	0.000	0.571
3G	W/N	27.10, n = 8	24.60, n = 7	0.613	23.0	0.411	0.125	0.732
	N/W	22.76, n = 8	23.02, n = 7	0.397	20.0	0.643	0.321	0.911
	N/R	24.63, n = 8	26.19, n = 7	0.867	26.0	0.536	0.232	0.839
	R/N	20.93, n = 8	24.17, n = 7	0.463	21.0	0.625	0.304	0.902
	R/W	5.66, n = 8	2.36, n = 7	0.021	8.0	0.143	0.000	0.375
3H	W/N	33.52, n = 8	41.46, n = 7	0.002	3.0	0.946	0.786	1.000
	N/W	25.53, n = 8	38.36, n = 7	0.021	8.0	0.857	0.571	1.000
	N/R	20.85, n = 8	9.65, n = 7	0.009	6.0	0.107	0.000	0.375
	R/N	13.93, n = 8	6.62, n = 7	0.006	5.0	0.089	0.000	0.286
	R/W	6.83, n = 8	5.48, n = 7	0.613	23.0	0.411	0.125	0.732
4A	NREMS	21.24, n = 8	9.41, n = 7	0.021	8.0	0.143	0.000	0.375
4B	REMS	63.14, n = 8	33.23, n = 7	0.029	9.0	0.161	0.000	0.429
6C		2261, n = 8	2581, n = 7	0.160	15.5	0.723	0.411	0.964
6D		1704, n = 8	641, n = 7	0.002	3.0	0.054	0.000	0.196
6E		5.290, n = 8	5.617, n = 7	0.072	12.0	0.786	0.500	1.000
6F		5.885, n = 8	6.246, n = 7	0.189	16.0	0.714	0.411	0.964
6G		1.972, n = 8	1.772, n = 7	0.054	11.0	0.196	0.000	0.500
6H		2.046, n = 8	1.810, n = 7	0.054	11.0	0.196	0.000	0.464

### Sleep architecture and fragmentation of S1 mice differ from sleep/wake patterns of BL6 mice

**Figure 3:**
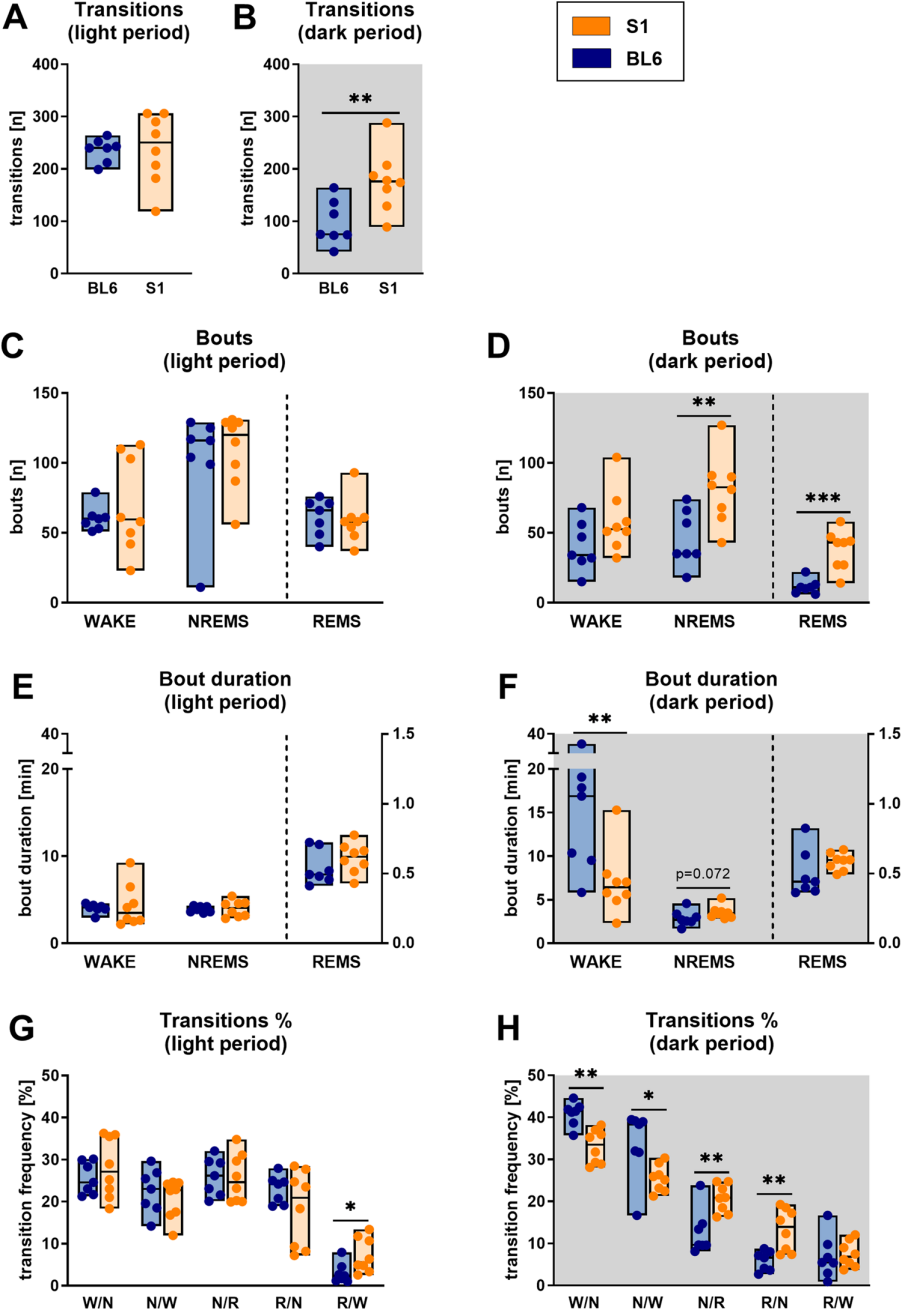
Sleep architecture and fragmentation in S1 (n = 8) compared to BL6 mice (n = 7). Total numbers of transitions were higher in S1 mice in the dark [(**A**) p = 0.009], but not the light period [(**B**) p = 0.758]. In the light period, bout numbers were comparable to those of BL6 mice [(**C)** WAKE: p = 0.979; NREMS: p = 0.676; REMS: p = 0.515]. However, S1 mice showed increased numbers of NREMS (p = 0.009) and REMS (p < 0.001), but not WAKE bouts (p = 0.127) in the dark period (**D**). Bout durations during the light period were also similar to those of BL6 controls [(**E**) WAKE: p = 0.613; NREMS: p = 0.955; REMS: p = 0.336]. In the active phase (**F**), WAKE bout lengths were significantly decreased in S1 animals (p = 0.009), while there was only a non-significant trend towards longer NREMS bouts (p = 0.072) and no difference in REMS bout lengths (p = 0.121). The percentage of all transitions between WAKE and NREMS and NREMS and REMS were similar in S1 and BL6 mice (W/N: p = 0.613; N/W: p = 0.397; N/R: p = 0.867; R/N: p = 0.463) in the light period (**G**), only the percentage of awakenings from REMS was increased in S1 mice (R/W: p = 0.021). In contrast, S1 mice showed less transitions between WAKE and NREMS (W/N: p = 0.002; N/W: p = 0.021) in the dark period (**H**) while they transitioned more often between NREMS and REMS (N/R: p = 0.009; R/N: p = 0.006) and had similar percentage of REMS-to-WAKE transitions (R/W: p = 0.613) as compared to BL6 mice. All floating bars show min-to-max values with median and individual data points. Statistical significance was determined with a Mann–Whitney U test (*p < 0.05, **p < 0.01).

**Figure 4:**
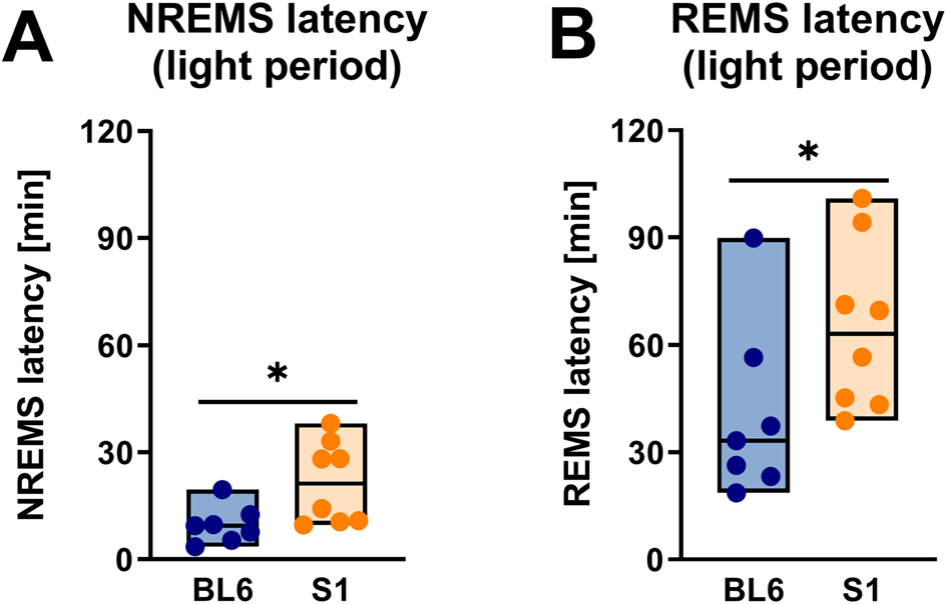
Sleep latencies at the onset of the light period in S1 (n = 8) and BL6 mice (n = 7). The latency to NREMS at the beginning of the light period was higher in S1 than BL6 mice [(**A**) p = 0.021]. Accordingly, also the time to enter REMS was increased in S1 mice [(**B**) p = 0.029]. All floating bars show min-to-max values with median and individual data points. Statistical significance was determined with a Mann–Whitney U test (*p < 0.05).

Also differences in sleep architecture and fragmentation (Fig. 3, for detailed statistics see Table 2) between both mouse strains were most noticeable in the dark (active) period (Fig. 3B,D,F,H), analogous to the quantitative changes in vigilance state distributions. In the nighttime, S1 mice showed strongly fragmented sleep/wake patterns, where periods of wakefulness were frequently interrupted by sleep intrusions. This was reflected by a significantly higher overall amount of vigilance state transitions (Fig. 3B, 176 vs. 75, p = 0.009) and increased numbers of NREMS and REMS bouts (Fig. 3D, NREMS: 83 vs. 35, p = 0.009; REMS: 43 vs. 11, p < 0.001). NREMS bouts in the dark period (Fig. 3F) were longer in S1 than BL6 mice, but while the difference was not statistically significant (p = 0.072), the AUC of 0.21 [0.00; 0.50] indicated a strong effect of strain. REMS bout lengths in the active phase (Fig. 3F) were similar in both mouse strains (p = 0.121). The amount of WAKE bouts in the dark period (Fig. 3D) was not significantly different (p = 0.127) in S1 and BL6 animals, but WAKE bout duration (Fig. 3F) was decreased (6.4 min vs. 16.9 min, p = 0.009). During the light period, on the other hand, total numbers of transitions (Fig. 3A, p = 0.758), bout numbers (Fig. 3C, WAKE: p = 0.979; NREMS: p = 0.232; REMS: p = 0.463) and bout durations (Fig. 3E, WAKE: p = 0.613; NREMS: p = 0.955; REMS: p = 0.336) were comparable between both mouse strains. Percentage distributions of transitions between vigilance states were also significantly altered in S1 mice (Fig. 3G,H). While they showed significantly less WAKE-to-NREMS (W/N: p = 0.002) and NREMS-to-WAKE (N/W: p = 0.021) transitions in the dark period (Fig. 3H), the percentages of transitions between NREMS and REMS were increased (N/R: p = 0.009; R/N: p = 0.006). Percentages of REMS-to-WAKE transitions, however, were comparable to those of BL6 mice (R/W: p = 0.613). In the light period, the mouse strains showed no significant difference in percentages of transitions (Fig. 3G) between WAKE and NREMS (W/N: p = 0.613; N/W: p = 0.397) and also NREMS and REMS (N/R: p = 0.867; R/N: p = 0.463), but the percentage of awakenings from REMS was higher in S1 than BL6 mice (R/W: 5.7% vs. 2.4%, p = 0.021). Interestingly, also sleep latency at the beginning of the inactive period was increased in S1 mice (Fig. 4). On average, they took significantly longer than BL6 mice (21.2 min vs. 9.4 min, p = 0.021) to enter the first episodes of NREMS (Fig. 4A). As a consequence, also the latency to REMS (Fig. 4B) was increased (63.1 min vs. 33.2 min, p = 0.029). S1 mice continued to show coherent REMS episodes after the light-to-dark transition (Supplementary Fig. S2), while BL6 controls did not show any REMS before about 6.5 h into the active phase (103.1 min vs. 398.8 min, p = 0.014).

### Power spectral densities in specific frequency bands are altered in S1 compared to BL6 mice across all vigilance states

**Figure 5:**
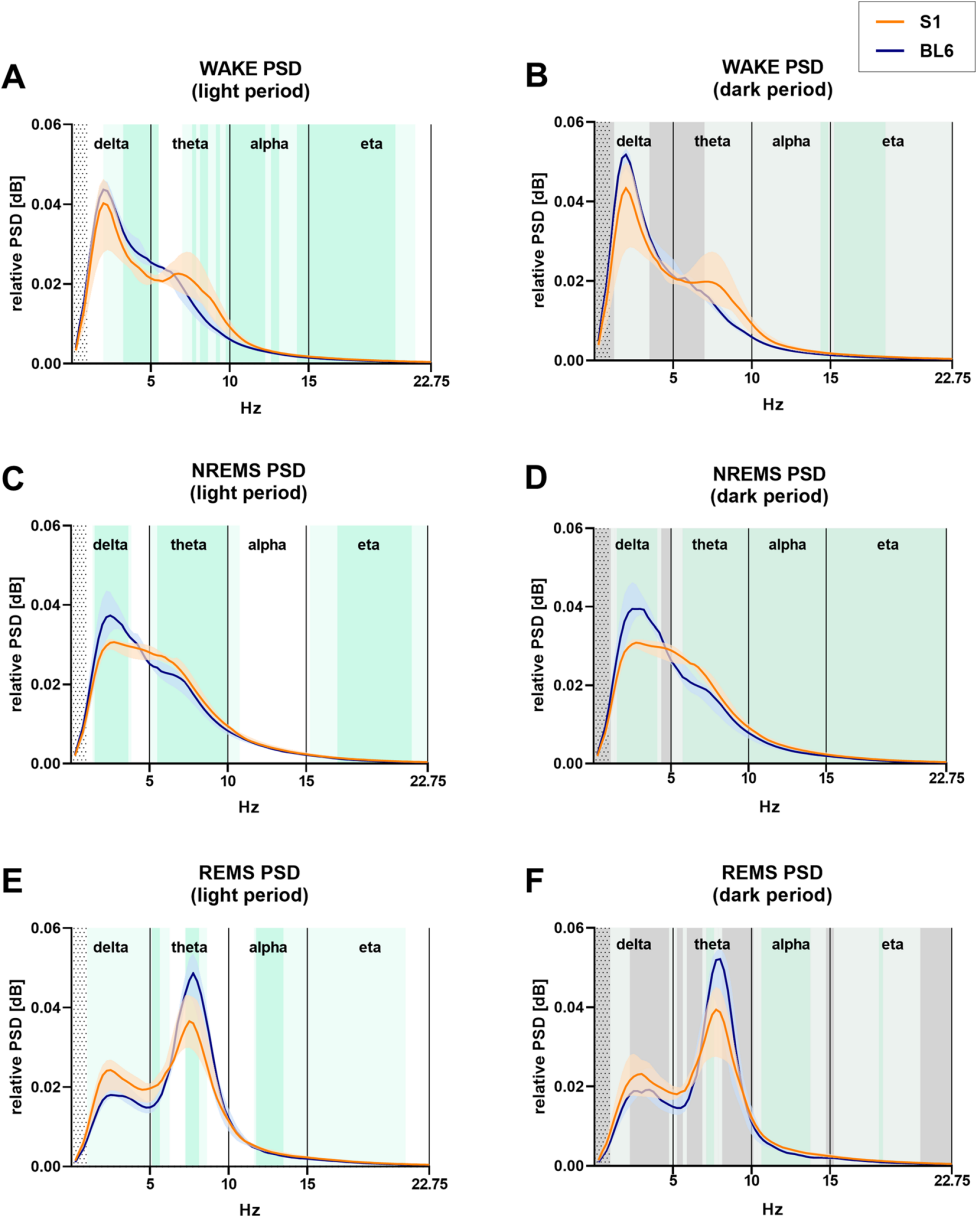
Power spectral density (PSD) in the 1–22.75 Hz frequency range (0.25 Hz bins) for each vigilance state in S1 (n = 6) and BL6 (n = 7) mice. During wakefulness in the light period (**A**), S1 mice showed lower power in the delta band (3.25–5.50 Hz), but significantly increased PSD in the high theta (7.75 Hz, 8.25–8.50 Hz, 9.25 Hz), alpha (9.75–12.25 Hz, 12.75–13.00 Hz, 14.25–15.00 Hz) and eta ranges (15.00–20.50 Hz). In the dark phase (**B**), WAKE eta power was higher in S1 than BL6 mice (15.25–18.25 Hz). S1 mice showed less power in the delta band [(**C**) 1.75–3.50 Hz; (**D**) 1.75–4.00 Hz] during NREMS, while power in the theta range was increased [(**C**) 5.50–10.00 Hz; (**D**) 5.75–10.00 Hz]. Also, NREMS alpha and eta power [(**C**) 17.00–21.75 Hz; (**D**) 10.00–22.75 Hz] were higher in S1 than BL6 mice. The for REMS characteristic power peak around 8 Hz was decreased in S1 versus BL6 mice in light [(**E**) 7.25–8.00 Hz] and dark period [(**F**) 7.25–7.50 Hz]. Power in the alpha frequency band, however, was higher in S1 mice [(**E**) 12.00–13.50 Hz; (**F**) 10.75–13.75 Hz]. All graphs show median PSD values in 0.25 Hz steps as lines plus shaded areas for interquartile ranges. Dotted areas indicate where a 1 Hz high pass filter was applied, dotted lines separate delta, theta, alpha and eta frequency bands. Green shaded areas indicate that the CI did not include 0.5, i.e. statistical significance, light green shades indicate AUC values > 0.7; i.e. relevant effects.

A spectral EEG analysis revealed significant differences between PSD in S1 and BL6 mouse strains for all vigilance states in the 1–22.75 Hz range (Fig. 5, for a more detailed overview of corresponding AUC values please see Supplementary Fig. S3). Differences were also detectable for EEG band powers (Supplementary Fig. S4, for corresponding statistics please see Supplementary Table S1). In the WAKE state (Fig. 5A,B), spectral power alterations in S1 mice concerned different frequency ranges in light and dark period. In the inactive phase (Fig. 5A and Supplementary Fig. S4A), S1 mice showed significantly lower power at specific frequencies in the high delta/low theta range, but increased power in the high theta, alpha and eta frequency bands. During the dark phase (Fig. 5B and Supplementary Fig. S4B), WAKE PSD was increased in S1 mice compared to BL6 mice in high alpha and eta frequencies. Also, in light and dark period NREMS (Fig. 5C,D and Supplementary Fig. S4C,D), the PSD differed significantly between S1 and BL6 mice. Here, S1 mice showed less power in the delta slow wave range, while theta power was increased. Similar to WAKE, PSD in the higher frequency ranges was increased in S1 mice during NREMS episodes in the light period (Fig. 5C and Supplementary Fig. S4C) and even more in the dark period (Fig. 5D and Supplementary Fig. S4D). The 8 Hz power peak, which is characteristic for REMS, was attenuated in S1 mice in active and inactive phase (Fig. 5E,F). Moreover, also during REMS, S1 mice showed increased alpha band power in comparison to BL6 mice, which was more pronounced in the dark (Fig. 5F and Supplementary Fig. S4F) than the light period (Fig. 5E and Supplementary Fig. S4E).

### Sleep spindle amounts, density and durations differ between S1 and BL6 mice

**Figure 6:**
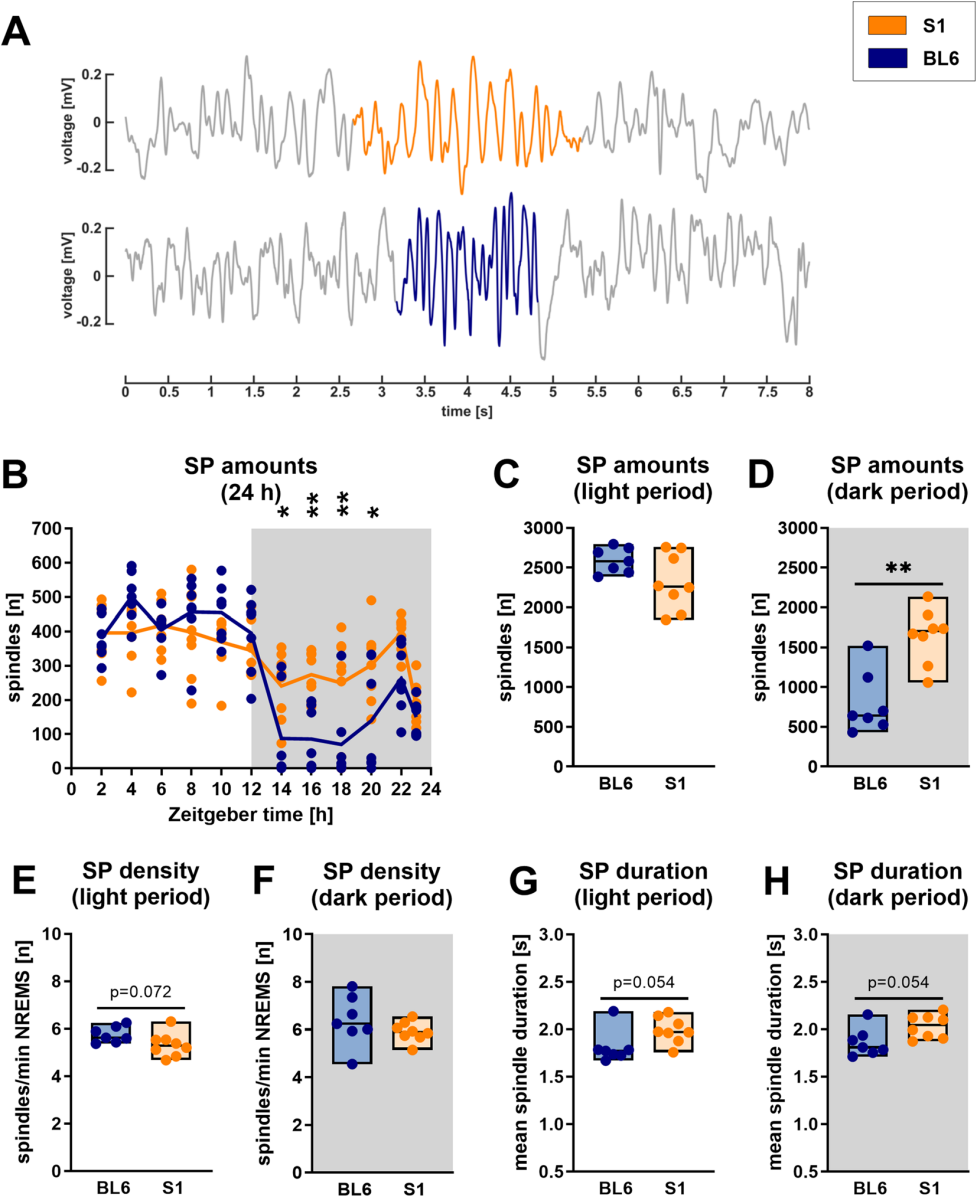
Sleep spindle (SP) detection in S1 (n = 8) and BL6 (n = 7) mice. Corresponding to increased NREMS percentages (Fig. 2B), also SP amounts were significantly higher in S1 than BL6 mice in the dark period [(**B**) ‘strain’: p = 0.067, ‘time’: p < 0.001, ‘time × strain interaction’: p < 0.001; (**D**) p = 0.002]. The number of SP per minute of NREMS, however, was unaltered [(**F**) p = 0.189]. In the light period, SP amounts did not differ between S1 and BL6 mice [(**C**) p = 0.160], while a trend towards higher SP density was observed [(**E**) p = 0.072]. Average SP duration, however, was (non-significantly) shorter in S1 than BL6 mice [(**G**,**H**) p = 0.054]. (**A**) Representative examples of sleep spindles detected in raw EEG traces of a S1 and a BL6 mouse. (**B**) The graph shows 2 h means as lines plus individual data points, white and grey backgrounds indicate light and dark period. Statistical significance was determined by two-way RM ANOVA with one-factor repetition (Sidak post-hoc test). (**C**–**H**) All floating bars show min-to-max values with median and individual data points. Statistical significance was determined with a Mann–Whitney U test (*p < 0.05, **p < 0.01).

Lastly, we also compared different features of NREMS sleep spindles (SP) in S1 and BL6 mice (Fig. 6, for detailed statistics refer to Tables 1 and 2 and Supplementary Fig. S1). S1 mice showed significantly higher spindle amounts (1704 SP vs. 641 SP, p = 0.002) during the dark period (Fig. 6D), while the circadian distribution was in line with the increased time they spent in NREMS (Fig. 6B, ‘strain’: p = 0.067, ‘time x strain’: p < 0.001). The number of spindles per minute of NREMS in the dark period (Fig. 6F) was unaltered (p = 0.189). In the light period, spindle amounts (Fig. 6C) were comparable between both groups (p = 0.160), however, we observed a non-significant trend (p = 0.072) towards a decrease in spindle density (Fig. 6E) in S1 mice, with an AUC of 0.79 [0.50; 1.00], indicating a strong effect of strain. Spindle durations (Fig. 6G,H) were increased in S1 mice across the whole recording period (AUC = 0.20 [0.00; 0.50], strong effect of strain), although the effect did not reach statistical significance (p = 0.054).

## Discussion

Disturbed sleep has been proposed as a possible confounding factor in the process of fear extinction and, thus, a risk factor for anxiety- and trauma-related disorders^29,31,54,55^. To investigate whether the S1 mouse strain, for which a severe impairment of fear extinction learning has been previously described by different groups^34,36,37,41–43,56^, shows aberrant sleep patterns at baseline, we examined circadian sleep/wake behaviors of S1 mice in comparison to those of typically extinction-competent BL6 mice. Within the present study, we uncovered profound differences between both mouse strains regarding the diurnal distribution of sleep and wakefulness, but also sleep architecture, spectral EEG features and sleep spindle events. Below, we summarize our main findings in the S1 mouse strain and discuss them in the context of findings from anxiety and PTSD patients, but also other rodent models of anxiety- and trauma-related disorders. Furthermore, we hypothesize how these altered sleep features could potentially predispose this mouse strain for maladaptive fear processing. These new findings and ideas are laying a foundation for closer investigation of causal relationships in future studies.

### Hypersomnia-like features: sleep intrusions in the dark period

During the active period, S1 mice showed increased sleep propensity, while they had difficulty in maintaining longer WAKE episodes. This may reflect a higher need for sleep to make up for a poorer sleep quality during the inactive period. From a human perspective, there are abundant reports of daytime sleepiness and poor daytime performance in anxiety and PTSD patients^57–59^. Of course, insomnia is a prominent symptom of PTSD and anxiety-related disorders^17,60^, however, it has been suggested that in anxiety disorders, hypersomnia may co-exist with insomnia, forming a vicious cycle of two opposing dimensions of sleep disturbance^61^. This combination of non-restorative sleep and impairment of daytime functioning may interfere with emotional coping, making an individual more vulnerable for the development and maintenance of anxiety or PTSD^16,29^. Interestingly, also in a genetic mouse model of high trait anxiety, hypersomniac sleep patterns were found, with frequent intrusions of NREMS-REMS bouts into WAKE episodes during the dark period^62^. On the other hand, many studies using classical fear conditioning as a rodent model for PTSD reported disrupted and reduced sleep, particularly REMS (for review see Ref.^63^), although also sleep increases or no changes were reported. This, however, does not compare to our baseline observations in behaviorally naïve S1 mice. Further experiments will be necessary to determine the effect of fear conditioning and extinction on sleep (and vice versa) in this mouse strain.

### Insomnia-like features: increased sleep latency and awakenings from REMS in the light period

Besides frequent intrusions of sleep episodes into wakefulness in the dark period, also sleep architecture in the light period was altered in S1 mice. There, they showed a significantly higher percentage of awakenings from REMS and also an increased latency to enter sleep as compared to BL6 mice. This may correspond to insomnia symptoms (i.e. difficulties falling and staying asleep) which are frequently reported by anxiety and PTSD patients^17,60^. Studies in humans reported a delayed sleep onset in generalized anxiety disorder^64^ and panic disorder^20,65^. In PTSD patients, one study associated longer sleep onset latency with more severe fear reinstatement and PTSD symptomatology^66^. The occurrence of insomnia was even proposed as a predictor of anxiety and other mental disorders^24^. Interestingly, in several clinical trials, targeted treatment of insomnia and nightmares led to an improvement of overall PTSD symptomatology (e.g. Refs.^67,68^), which suggests a causal involvement in development and maintenance of PTSD. Generally, nightmares are described as disturbing dreams that typically occur during REMS and are followed by fearful awakening^69^. In this respect, our finding of increased awakenings from REMS in S1 mice is particularly interesting, although it is of course impossible to determine whether those REMS-WAKE episodes represent nightmare-like events. However, a high degree of (especially REM) sleep fragmentation and more frequent awakenings from REMS were described as polysomnographic features of anxiety disorders and PTSD^17,70,71^. REMS disruption has also been proposed as a central factor in the development of PTSD^72,73^. In line with human studies, experiments in BL6 mice showed that low REMS continuity at baseline was correlated with higher levels of hyperarousal one month after contextual fear conditioning^46^. Moreover, as a proof of principle, experimentally induced sleep fragmentation led to an increase of anxiety-like behavior in rats^74^. Another study found more spontaneous awakenings in more anxious BALBc/J mice than in BL6 controls^75^. In the light of this evidence, the observed alterations in sleep architecture could potentially predispose the S1 mouse strain towards maladaptive fear processing and increased anxiety-like behavior.

### Compromised sleep quality: reduced NREMS delta and REMS theta power

Additionally, several spectral features suggested a reduced overall sleep quality in S1 mice compared to BL6 mice. Most importantly, while during NREMS power in the slow wave range was reduced, in REMS the characteristic 8 Hz peak was attenuated. Reduced delta power during slow wave sleep (NREMS stage N3) was reported in healthy individuals with high trait anxiety^76^, but also in panic disorder and PTSD patients^77^. In a very recent study, time spent in N3 was significantly associated with lower morning anxiety in healthy participants, indicating that NREMS has an anxiolytic-like effect^78^. The amount of N3 sleep and higher delta power predicted greater re-engagement of the medial prefrontal cortex the next day, suggesting that the anxiolytic-like effect of slow wave sleep is mediated by overnight restoration of prefrontal activity and associated limbic functional connectivity relevant for the regulation of anxiety^78^. Although we are not aware of a method to reliably classify NREMS stages in mice, reduced power in the delta range could indicate that NREMS in S1 contains less (anxiolytic-like) slow wave sleep than in BL6 mice, while increased power in theta, alpha and eta bands may arise from lighter NREMS stages. From a homeostatic point of view, such a lack of deep NREMS sleep in the inactive period would also explain the frequent sleep intrusions in S1 mice in the active period. Moreover, it has been shown that the infralimbic cortex, which plays an important role in mediating successful extinction^79^, is hypoactivated in S1 mice during extinction memory retrieval^33,41,43^. This as well could be a consequence of decreased NREMS delta power and an associated loss of its restorative function.

In contrast to NREMS, the involvement of REMS and theta oscillatory activity in emotional memory processing is quite established^1,2^. During REMS, high activity in limbic structures may allow reactivation of previously acquired affective experiences while theta oscillations within subcortical and cortical structures serve as a carrier frequency and facilitate the consolidation of recently experienced emotional events and their integration in the context of pre-existing memories^1^. Like this, REMS may act as a form of 'overnight therapy', preserving the informational core of emotional experiences and yet de-potentiating their original emotional charge^1^. Interestingly, higher prefrontal theta power during REMS was found in resilient trauma victims versus individuals who developed PTSD and proposed as a biomarker for the capacity to process traumatic memories^80^. Furthermore, in a stress-induced mouse model of PTSD, a greater reduction in theta power during transitions to REMS following stress exposure was correlated with higher freezing levels during fear extinction recall^81^. Therefore, we hypothesize that in the S1 mouse strain a reduced activity in the corticolimbic circuitry^33,41,43^, together with reduced theta power during REMS, may hinder ‘night-time’ reactivation and subsequent integration of emotional memories (e.g. extinction memories) in the context of pre-existing information (e.g. fear memories, contextual information). Whether these spectral features indeed correlate with and contribute to the extinction-deficient phenotype described for the S1 mouse model will have to be demonstrated in further studies.

### Sleep spindle alterations: potential sleep biomarkers for anxiety- and trauma-related disorders?

In short, sleep spindles are burst-like EEG signals between 12 and 15 Hz, occurring within the thalamo-cortical circuitry during NREMS stages N1 or N2^82^. Interestingly, they were attributed a significant role in memory formation^83^, but only a few studies examined their role in the context of anxiety- and trauma-related disorders. For example, a study in healthy participants associated greater amounts of N2 sleep and more parietal spindles with fewer intrusive memories following the viewing of a traumatic film, while increased stage N1 NREMS and awakenings after sleep onset were associated with more intrusions^84^. To our knowledge, there is currently no study available that thoroughly investigated sleep spindle characteristics in a mouse model of PTSD or anxiety disorders. Compared to BL6 mice, we found increased amounts of spindles during the dark period (analogous to increased amounts of NREMS) in the S1 mouse strain and trends towards increased spindle durations and reduced spindle density in the inactive period. Since also in humans sleep spindle features have just begun to be explored as possible biomarkers for anxiety and trauma-related disorders, we want to refrain from an over-interpretation of our data. However, with regard to their proposed role in memory formation, sleep spindles should not be overlooked in future EEG studies in patients but also rodent models of anxiety disorders and PTSD.

### Limitations of our study and future directions

Our study has some limitations worth noting. Most importantly, we want to emphasize that the data presented here is purely descriptive and was not correlated with any behavioral tests. In the previous sections, we hypothesized how the identified alterations in baseline diurnal distribution of sleep and wakefulness, sleep architecture and quality could potentially predispose the S1 mouse strain towards maladaptive fear processing. In future experiments, however, it will be necessary to demonstrate a causal relationship between the uncovered sleep features and impaired fear extinction in the S1 mouse strain. This hypothesis could further be strengthened by EEG studies in other 129 sub-strains, for which an extinction-impaired phenotype has been described^34^. Moreover, it will be of interest whether particular sleep parameters change in the S1 or other 129 sub-strains in response to a fear conditioning/extinction paradigm.

Our data also indicate that the circadian clock function may be altered in S1 mice, as they display only a moderate diurnal rhythmicity compared to BL6 mice. This intriguing phenotype will have to be addressed in additional experiments investigating circadian rhythms and sleep homeostasis following sleep deprivation or in free-running conditions. The underlying cause of the described sleep/wake alterations remains unknown, however, mechanistic studies testing for mutations of clock genes or altered release of sleep regulatory substances (e.g. adenosine, melatonin, serotonin, dopamine) may help to not only understand the distinctive sleep/wake patterns of S1 mice, but also their behavioral phenotype. This is of particular relevance, given that disruptions of the circadian rhythm have been associated with psychiatric disorders^85^.

As a general limitation of sleep studies in rodents, it should be considered that the amount, circadian distribution and fragmentation of sleep strongly varies between species^86^. Unlike humans, nocturnal animals like rodents are predominantly asleep during the light period and, as polyphasic sleepers, show shorter NREMS-REMS cycles that also may occur during their active period^87^. However, we believe that, if interpreted with care, data from translational rodent models have the potential to aid research on the role of (disturbed) sleep in psychiatric conditions.

## Conclusion

In summary, our characterization of sleep/wake behaviors in the S1 mouse model of impaired fear extinction further strengthens its face validity with regard to sleep disturbances reported by anxiety and PTSD patients, such as daytime sleepiness and poor daytime performance, insomnia and compromised sleep quality. Using such rodent models as a platform to investigate potential sleep biomarkers for anxiety- and trauma-related disorders could facilitate the establishment of better diagnostic tools, protective measures (e.g. suitability tests for occupations with higher risk of traumatic exposure) and monitoring strategies for treatment responses. We identified several distinctive features in the sleep patterns of S1 mice that could potentially predispose this mouse strain towards the development of an anxiety-/PTSD-like phenotype, however, more studies will be necessary to demonstrate causality or correlation with a behavioral phenotype and unravel the underlying pathophysiological mechanisms. For future translational studies, it should also be considered that poor sleep may not only be a risk factor for the development of PTSD and anxiety-related disorders but might also interfere with treatment interventions, for example by disrupting fear extinction^14,15,88^. In this regard, it will be of great interest whether manipulating sleep before or after fear extinction could rescue or attenuate extinction-deficient and anxiety-like behaviors in the S1 mouse strain or other rodent models. This may provide further indication if and how clinically addressing (disturbed) sleep in patients before or after psychotherapeutic interventions could improve treatment outcomes and protect from a later relapse. Along these lines, a small study in patients with social anxiety disorder provided preliminary evidence that naps after exposure therapy enhance extinction learning^89^. Overall, genetic mouse models like the S1 mouse strain may hold the potential to uncover the role of disturbed sleep in anxiety-related disorders and PTSD and give deeper insights into the mechanistic underpinnings, for example in translational transcriptomics or genom-wide association studies (see e.g. Ref.^90^).

## Supplementary Information

Supplementary Information.

## Abbreviations

AUC: Area under the curve
BL6: C57BL/6
CI: Confidence interval
EEG: Electroencephalography/electroencephalogram
EMG: Electromyography/electromyogram
NREMS: Non-rapid eye movement sleep
PSD: Power spectral density
PTSD: Post-traumatic stress disorder
RDoC: Research domain criteria
REMS: Rapid-eye movement sleep
RMS: Root mean square
S1: 129S1/SvImJ
SP: Sleep spindle
WAKE: Wakefulness
