# Supplementary material for: Altered sleep behavior in a genetic mouse model of impaired fear extinction
Source: Sci Rep. 2021 Apr 26;11:8978. doi: 10.1038/s41598-021-88475-2 (PMC8076259; doi:10.1038/s41598-021-88475-2)
Supplement: Supplementary file 1 — Supplementary Information. [file 41598_2021_88475_MOESM1_ESM.docx]

# Supplementary Information

**Altered sleep behavior in a genetic mouse model of impaired fear extinction**

Eva Maria Fritz, Matthias Kreuzer, Alp Altunkaya, Nicolas Singewald, Thomas Fenzl

## Supplementary Figure S1


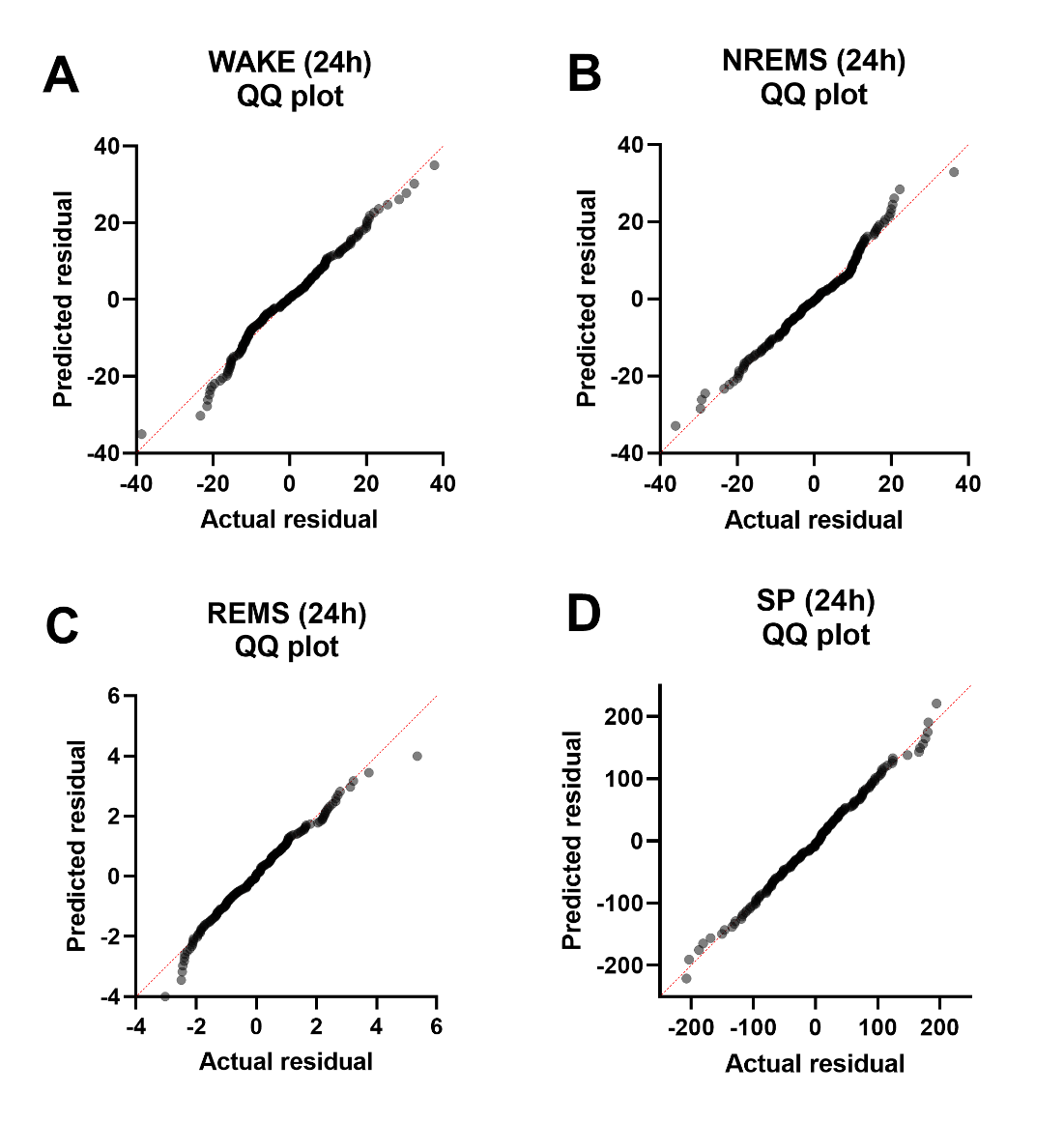


**Supplementary Figure 1. Quantile-quantile (QQ) plots of residuals for data of 24h sleep/wake behavior (Fig. 2A-B) and sleep spindles (Fig. 6A).** Visual assessment suggests a normal distribution, as most data points form a straight line that follows the line of identity. *Dotted red line shows the line of identity.*

## Supplementary Figure S2

**Supplementary Figure 2. Latencies to NREMS and REMS at the beginning of the active period in S1 (n=8) and BL6 (n=7) mice.** During their active phase, S1 and BL6 mice had similar latencies to the first NREMS episodes in the dark period (**A**, p=0.397). While S1 mice showed coherent REMS episodes not long after the transition from light to dark, BL6 mice did not enter REMS until about 6.5 h into the dark period (**B**, p=0.014). *All floating bars show min-to-max values with median and individual data points. Statistical significance was determined with a Mann-Whitney U test (*p<0.05).*

## *
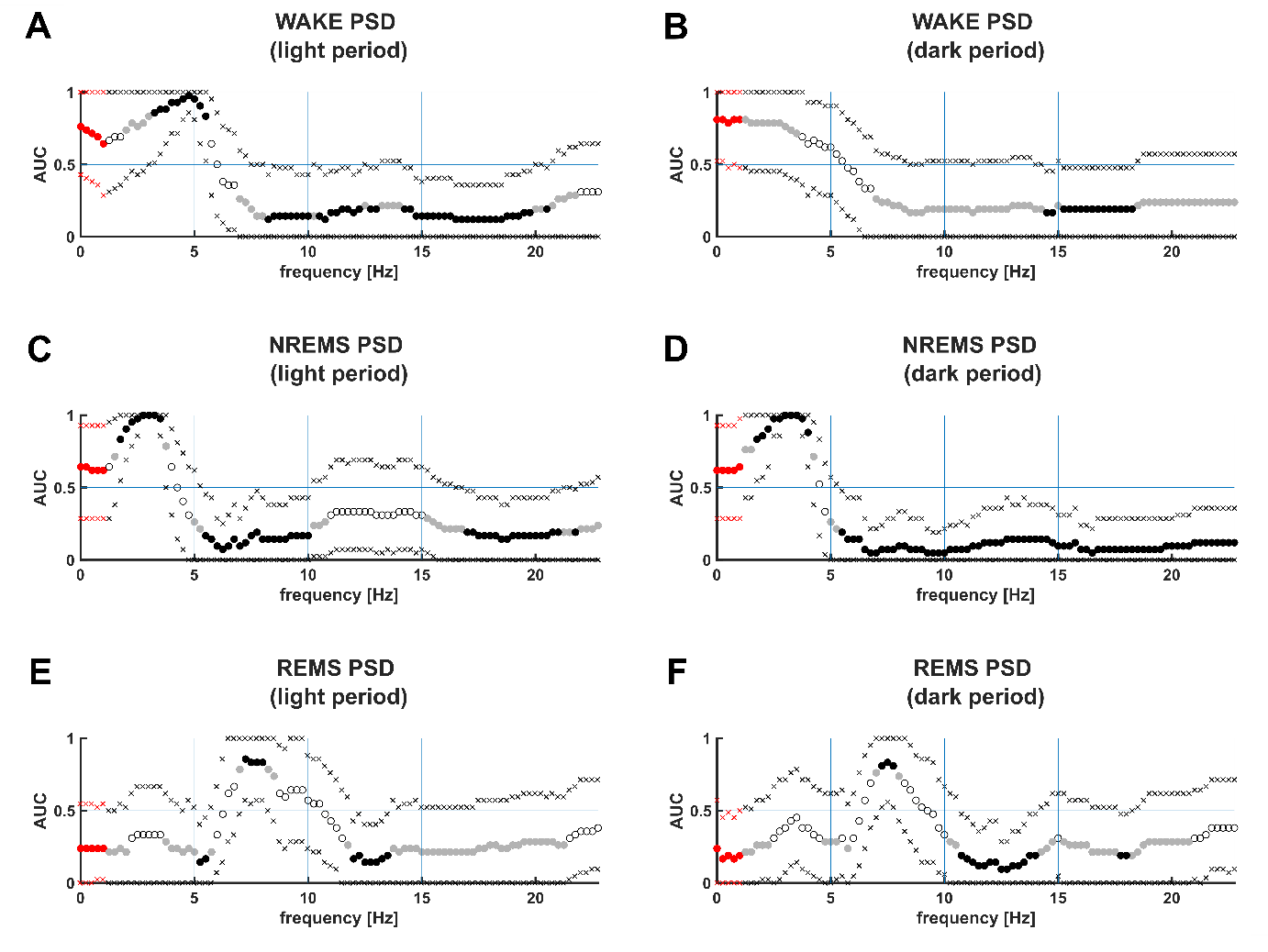
*Supplementary Figure S3

**Supplementary Figure 3. AUC with CI for PSD values (0.25 Hz bins) in the 1-22.75 Hz frequency range for each vigilance state in S1 (n=6) and BL6 (n=7) mice.** *Black dots indicate that the CI did not include 0.5, i.e. statistical significance, grey dots indicate AUC values >0.7; i.e. relevant effects. x marks upper and lower limits for 95% CIs. Red dots and x indicate where a 1 Hz high-pass filter was applied.*

## Supplementary Figure S4

**Supplementary Figure 4. PSD for defined frequency bands per vigilance state in light and dark period in S1 (n=6) and BL6 (n=7) mice.** In the WAKE state (**A, B**), S1 mice showed higher overall power in the alpha band (**A**, p=0.035) and in the eta band (**A**, p=0.022) during the light period. Delta power was decreased (**C**, p=0.014; **D**, p=0.002), while theta power was increased (**C**, p=0.005; **D**, p=0.005) in S1 vs. BL6 mice during NREMS episodes in light and dark period. In the dark period, also PSD in alpha (**D**, p=0.008) and eta bands (**C**, p=0.073; **D**, p=0.008) was higher in S1 mice. **E, F)** During REMS in the active phase, power in the alpha range was increased in S1 vs. BL6 mice (**F**, p=0.022). All floating bars show min-to-max values with median and individual data points. Statistical significance was determined with a Mann-Whitney U test (*p<0.05).

## Supplementary Table S1. Statistical results of Mann-Whitney U tests and corresponding AUC with 95% CI.

| **Figure** | | **Mann-Whitney U** | | | | **AUC** | | |
| --- | --- | --- | --- | --- | --- | --- | --- | --- |
|  |  | **median (S1)** | **median (BL6)** | **p** | **U** | **AUC** | **95% CI** | |
| **S1A** | NREMS | 49.36, n=8 | 107.3, n=7 | 0.397 | 20.0 | 0.643 | 0.321 | 0.911 |
|  |  |  |  |  |  |  |  |  |
| **S1B** | REMS | 103.1, n=8 | 398.8, n=7 | 0.014 | 7.0 | 0.875 | 0.625 | 1.000 |
|  |  |  |  |  |  |  |  |  |
| **S3A** | delta | 0.4968, n=6 | 0.5675, n=7 | 0.101 | 9.0 | 0.214 | 0.000 | 0.548 |
|  | theta | 0.2604, n=6 | 0.2119, n=7 | 0.138 | 10.0 | 0.762 | 0.429 | 1.000 |
|  | alpha | 0.0833, n=6 | 0.0666, n=7 | 0.035 | 6.0 | 0.857 | 0.571 | 1.000 |
|  | eta | 0.0242, n=6 | 0.0201, n=7 | 0.022 | 5.0 | 0.881 | 0.643 | 1.000 |
|  |  |  |  |  |  |  |  |  |
| **S3B** | delta | 0.5229, n=6 | 0.6054, n=7 | 0.101 | 9.0 | 0.214 | 0.000 | 0.548 |
|  | theta | 0.2386, n=6 | 0.1884, n=7 | 0.138 | 10.0 | 0.762 | 0.429 | 1.000 |
|  | alpha | 0.0824, n=6 | 0.0595, n=7 | 0.073 | 8.0 | 0.810 | 0.476 | 1.000 |
|  | eta | 0.0242, n=6 | 0.0177, n=7 | 0.138 | 10.0 | 0.762 | 0.429 | 1.000 |
|  |  |  |  |  |  |  |  |  |
| **S3C** | delta | 0.4567, n=6 | 0.5105, n=7 | 0.014 | 4.0 | 0.095 | 0.000 | 0.310 |
|  | theta | 0.2654, n=6 | 0.2440, n=7 | 0.005 | 2.0 | 0.952 | 0.810 | 1.000 |
|  | alpha | 0.1008, n=6 | 0.0956, n=7 | 0.295 | 13.0 | 0.691 | 0.357 | 0.929 |
|  | eta | 0.0262, n=6 | 0.0223, n=7 | 0.073 | 8.0 | 0.810 | 0.524 | 1.000 |
|  |  |  |  |  |  |  |  |  |
| **S3D** | delta | 0.4603, n=6 | 0.5431, n=7 | 0.002 | 1.0 | 0.024 | 0.000 | 0.143 |
|  | theta | 0.2659, n=6 | 0.2270, n=7 | 0.005 | 2.0 | 0.952 | 0.786 | 1.000 |
|  | alpha | 0.1004, n=6 | 0.0841, n=7 | 0.008 | 3.0 | 0.929 | 0.714 | 1.000 |
|  | eta | 0.0264, n=6 | 0.0207, n=7 | 0.008 | 3.0 | 0.929 | 0.714 | 1.000 |
|  |  |  |  |  |  |  |  |  |
| **S3E** | delta | 0.3373, n=6 | 0.2555, n=7 | 0.234 | 12.0 | 0.714 | 0.405 | 1.000 |
|  | theta | 0.3926, n=6 | 0.4788, n=7 | 0.101 | 9.0 | 0.214 | 0.000 | 0.524 |
|  | alpha | 0.1035, n=6 | 0.0947, n=7 | 0.366 | 14.0 | 0.667 | 0.333 | 0.952 |
|  | eta | 0.0282, n=6 | 0.0238, n=7 | 0.181 | 11.0 | 0.738 | 0.429 | 1.000 |
|  |  |  |  |  |  |  |  |  |
| **S3F** | delta | 0.3263, n=6 | 0.2673, n=7 | 0.181 | 11.0 | 0.738 | 0.429 | 0.976 |
|  | theta | 0.3953, n=6 | 0.4795, n=7 | 0.101 | 9.0 | 0.214 | 0.000 | 0.524 |
|  | alpha | 0.1033, n=6 | 0.0882, n=7 | 0.022 | 5.0 | 0.881 | 0.643 | 1.000 |
|  | eta | 0.0298, n=6 | 0.0234, n=7 | 0.181 | 11.0 | 0.738 | 0.429 | 1.000 |
|  |  |  |  |  |  |  |  |  |
